# Heat shock factor 1 is inactivated by amino acid deprivation

**DOI:** 10.1007/s12192-012-0347-1

**Published:** 2012-07-14

**Authors:** Sanne M. M. Hensen, Lonneke Heldens, Chrissy M. W. van Enckevort, Siebe T. van Genesen, Ger J. M. Pruijn, Nicolette H. Lubsen

**Affiliations:** Department of Biomolecular Chemistry, Radboud University Nijmegen, P.O. Box 9101, 6500 HB Nijmegen, The Netherlands

**Keywords:** Heat shock factor 1, Amino acid deprivation, HSPA1A, Stress response

## Abstract

Mammalian cells respond to a lack of amino acids by activating a transcriptional program with the transcription factor ATF4 as one of the main actors. When cells are faced with cytoplasmic proteotoxic stress, a quite different transcriptional response is mounted, the heat shock response, which is mediated by HSF1. Here, we show that amino acid deprivation results in the inactivation of HSF1. In amino acid deprived cells, active HSF1 loses its DNA binding activity as demonstrated by EMSA and ChIP. A sharp decrease in the transcript level of HSF1 target genes such as HSPA1A (Hsp70), DNAJB1 (Hsp40), and HSP90AA1 is also seen. HSPA1A mRNA, but not DNAJB1 mRNA, was also destabilized. In cells cultured with limiting leucine, HSF1 activity also declined. Lack of amino acids thus could lead to a lower chaperoning capacity and cellular frailty. We show that the nutrient sensing response unit of the ASNS gene contains an HSF1 binding site, but we could not detect binding of HSF1 to this site in vivo. Expression of either an HSF1 mutant lacking the activation domain (HSF379) or an HSF1 mutant unable to bind DNA (K80Q) had only a minor effect on the transcript levels of amino acid deprivation responsive genes.

**Electronic supplementary material:**

The online version of this article (doi:10.1007/s12192-012-0347-1) contains supplementary material, which is available to authorized users.

## Introduction

All cells have a number of distinct programmed responses to cope with various adverse conditions. In eukaryotic cells, lack of amino acids evokes a deceptively simple initial response. Accumulation of uncharged tRNAs activates general control non-derepressible 2 kinase (GCN2) (Krishnamoorthy et al. 2001) which phosphorylates the eukaryotic translation initiation factor 2α (eIF2α). This results in a general inhibition of protein synthesis and in the selective translation of a few mRNAs amongst which that encoding the transcription factor ATF4 (reviewed in Wek et al. 2006). ATF4 then activates promoters by binding to the amino acid response element (AARE; Harding et al. 2000; Averous et al. 2004) or the nutrient sensing response unit (NSRU; Siu et al. 2002; Zhong et al. 2003) and thereby initiates a complex transcriptional program. Two well-known targets of ATF4 are the asparagine synthetase (ASNS) and the CHOP (DDIT3) promoters, which are the paradigms for the amino acid deprivation response. The activation of these promoters during the amino acid deprivation response has been dissected by Chen and co-workers (Chen et al. 2004) and Bruhat and co-workers (Bruhat et al. 2007). The binding of ATF4 closely correlates with the transcriptional activation. Later C/EBPβ and/or ATF3, also targets of ATF4, bind and repress ASNS promoter activity. The activation of the CHOP promoter requires not only ATF4 but also ATF2, which is constitutively present (Averous et al. 2004; Bruhat et al. 2000).

The amino acid deprivation response shows some overlap with the unfolded protein response (UPR) elicited by unfolding proteins accumulating in the ER. One branch of the UPR, PERK, is an eIF2α kinase and activation of the UPR thus also leads to eIF2α phosphorylation and the selective synthesis of ATF4 (reviewed in Holcik and Sonenberg 2005). ATF4 then activates some, but not all (see for example Gjymishka et al. 2008), of the promoters also activated by the amino acid deprivation response and in addition activates the promoters of genes encoding ER resident proteins. The distinction between the transcriptional program initiated by ATF4 as part of the UPR and that as part of the amino acid response is presumably dictated by auxiliary factors and heteromeric partners.

A third stress response that leads to eIF2α phosphorylation, this time by the PKR and HRI kinases, is that elicited by unfolding proteins in the cytoplasm, the heat shock response. Although ATF4 protein levels are increased upon heat stress and prototypical ATF4 target genes such as *ASNS* and *CHOP* are about 2-fold upregulated during heat shock (see for example the microarray data presented by Page et al. 2006), ATF4 is not thought to play a significant role in the heat shock response. The main actor in this response is heat shock factor 1 (HSF1), which upon stress is phosphorylated and translocated to the nucleus, where it activates the transcription of a number of genes mostly encoding heat shock proteins (reviewed in Wu 1995; Morimoto 1998; Voellmy 2004). These heat shock proteins act as chaperones for unfolded nuclear and cytosolic proteins, either refolding them or targeting them for degradation.

To date, little is known about the interaction between the heat shock response and the amino acid response. Xie et al. (2002) described that under stress conditions HSF1 physically interacts with C/EBPβ, one of the transcription factors involved in the amino acid response. We demonstrate that during leucine deprivation, and also during starvation for lysine or glutamine, nuclear HSF1 loses its DNA binding activity. HSPA1A mRNA is also destabilized (see also Eliasen et al. 2006a). We found that the NSRU of the ASNS promoter does contain an HSE, but we could not detect binding to this HSE in vivo. HSF1 did not appear to play a major role in the transcriptional response to amino acid deprivation as evidenced by the changes in transcript levels of amino acid deprivation responsive genes in cells stably expressing either an HSF1 mutant lacking the activation domains or an HSF1 mutant incapable of binding DNA. The physiological role of the inactivation of HSF1 during the amino acid response is thus not clear.

## Materials and methods

### Recombinant DNA constructs

The reporter plasmid pGL3-NSRU containing the nutrient sensing response unit (NSRU) was made by annealing the NSRU primers NSRU_fwd and NSRU_rev and cloning the double stranded oligonucleotide into the NheI and XhoI sites of pGL3 promoter (Promega). pGL3-NSRU1xmut and pGL3-NSRU2xmut were made as the pGL3-NSRU, using the corresponding oligonucleotides. Expression plasmid pcDNA5-HSF1 was made by inserting the Sfo/XhoI fragment of pOTB7-hHSF1 (Imagenes, www.imagenes-bio.de) containing the code for the C-terminal region of HSF1 in pcDNA5-HSF379 (dnHSF1) (Heldens et al. 2010). The pcDNA5-wtHSF1 (silent mutation) and the pcDNA5-HSF1K80Q mutant were made by performing site-directed mutagenesis on pcDNA5-HSF1 with respectively the HSF1_sil.mut and the HSF1_K80Q primers. Primers are listed in Table 1. All constructs were sequence verified.

**Table 1:** Primers

	Primer name	Primer sequence (5′ → 3′)
Cloning	NSRU_fwd	ctagcgcatgatgaaacttcccgcacgcgttacaggagcatgatgaaacttcccgcacgcgttacaggag
	NSRU_rev	tcgactcctgtaacgcgtgcgggaagtttcatcatgctcctgtaacgcgtgcgggaagtttcatcatgcg
	NSRU_1xmut_fwd	ctagcgcatgatgaaacaacccgcacgcgttacaggagcatgatgaaacttcccgcacgcgttacaggag
	NSRU_1xmut_rev	tcgactcctgtaacgcgtgcgggaagtttcatcatgctcctgtaacgcgtgcgggttgtttcatcatgcg
	NSRU_2xmut_fwd	ctagcgcatgatgaaacaacccgcacgcgttacaggagcatgatgaaacaacccgcacgcgttacaggag
	NSRU_2xmut_rev	tcgactcctgtaacgcgtgcgggttgtttcatcatgctcctgtaacgcgtgcgggttgtttcatcatgcg
	HSF1_sil.mut	cagaaagtcgtcaacaagcttatccagttcctgatctcactg
	HSF1_K80Q	catgtatggcttccggcaagtggtccacatcgagc
EMSA	HSE_EMSA_fwd	aacgagaatcttcgagaatggct
	HSE_EMSA_rev	agccattctcgaagattctcgtt
	NSRU_EMSA_fwd	gcaggcatgatgaaacttcccgcacgcgttacaggagccag
	NSRU_EMSA_rev	ctggctcctgtaacgcgtgcgggaagtttcatcatgcctgc
	2xNSRU_fwd	ctagcgcatgatgaaacttcccgcacgcgttacaggagcatgatgaaacttcccgcacgcgttacaggag
	2xNSRU_rev	tcgactcctgtaacgcgtgcgggaagtttcatcatgctcctgtaacgcgtgcgggaagtttcatcatgcg
	2xNSRUmut_fwd	ctagcgcatgatgaaacaacccgcacgcgttacaggagcatgatgaaacaacccgcacgcgttacaggag
	2xNSRUmut_rev	tcgactcctgtaacgcgtgcgggttgtttcatcatgctcctgtaacgcgtgcgggttgtttcatcatgcg
ChIP	ASNS_fwd	tggttggtcctcgcaggcat
	ASNS_rev	cgcttataccgacctggctcct
	DNAJB1_fwd	ggatgtcgcgtgtcgctgaa
	DNAJB1_ rev	cgaccagtcccggactctata
QPCR	GAPDH_fwd	ttccccatggtgtctgagc
	GAPDH_rev	atcttcttttgcgtcgccag
	ASNS_fwd	gcagctgaaagaagcccaagt
	ASNS_rev	tgtcttccatgccaattgca
	HSPA1A_fwd	ccgagaaggacgagtttgag
	HSPA1A_rev	acaaaaacagcaatcttggaaagg
	DNAJB1_fwd	ttccccagacatcaagaacc
	DNAJB1_rev	accctctcatggtccacaac
	HSP90_fwd	gttggtcctgtgcggtcact
	HSP90 _rev	tgggcaatttctgcctgaa

### Tissue culture

Flp-In T-REx-293 cells (Invitrogen) were manipulated according to the manufacturer’s instructions using the T-REx system (Invitrogen) to generate the stable cell lines HEK-HSF1K80Q and HEK-wtHSF1 that carry a single copy of the tetracycline-inducible plasmids pcDNA5-HSF1K80Q and pcDNA5-wtHSF1, respectively. T-REx HEK293-pcDNA5 and HEK-HSF379 (dnHSF1) were generated as described before (Heldens et al. 2010). The cells were cultured at 37°C/5% CO_2_ in high glucose DMEM medium supplemented with 10% fetal calf serum,100 U/ml penicillin, and 100 μg/ml streptomycin. Blasticidin (1.65 μg/ml; Invitrogen) and 100 μg/ml hygromycin were also added to the culture medium during maintenance of the cell lines, but were omitted during experiments. For amino acid starvation experiments, cells were washed with PBS and subsequently DMEM/F12 medium (Sigma) with or without leucine, glutamine, or lysine, supplemented with 10% dialyzed fetal calf serum, was added for the indicated times.

### Transfections and reporter gene assays

HEK293 cells were transiently transfected using Fugene-6 (Roche). Cells were seeded on 24-well plates and on the next day transfected with 0.2 μg plasmid per well: 20 ng pCMV-β-galactosidase and 180 ng luciferase reporter plasmid. Cells were harvested for reporter gene analysis at the time and under the culture conditions indicated. Cells were lysed in 200 μl reporter lysis mix (25 mM Bicine, 0.05% Tween 20, 0.05% Tween 80) for 10 min. For the β-galactosidase assay, 10 μl cell lysate was mixed with 100 μl Galacton solution [100 mM Na-phosphate pH 8.2, 5 mM MgCl_2_, 1% Galacton-Plus (Tropix)]. After 30-min incubation at room temperature, 150 μl accelerator II (Tropix) was added and luminescence was measured with the Lumat LB 9507 tube luminometer (Berthold). For the luciferase assay, 10 μl cell lysate was mixed with 50 μl luciferin solution and luminescence was again measured with the Lumat luminometer. All reporter gene assays were performed in triplicate. The activities of the reporter genes were corrected for transfection efficiency on basis of the β-galactosidase activity. Two-tailed Student’s *t* tests were performed to calculate the significance of the data.

### Western blot analysis

Cells were harvested in lysis buffer [25 mM Tris–HCl pH 7.5, 100 mM KCl, 1 mM DTE, 2 mM EDTA, 0.5 mM PMSF, 0.05% NP-40, 1× PhosSTOP (Roche), 1× protease inhibitor cocktail (Complete Mini, Roche)] and protein concentration was determined using a Bradford protein assay (Bio-Rad). For analysis of cytoplasmic and nuclear fractions, extracts were prepared using NE-per nuclear and cytoplasmic reagents (Pierce). Next, 4× sample buffer (200 mM Tris–HCl pH 6.8, 20% β-mercaptoethanol, 8% SDS, 40% glycerol, and 0.4% bromophenol blue) was added and the lysates were incubated at 95°C for 5 min. Protein samples were separated on a 10% SDS–polyacrylamide gel and transferred to nitrocellulose transfer membrane (Protran). For western blot analysis, the following antibodies were used: mouse monoclonal β-actin antibody (AC-15; Sigma; 1:5,000), rabbit polyclonal HSF1 antibody (SPA-901; Stressgen; 1:1,000), rabbit polyclonal DNAJB1 antibody (anti-Hsp40; SPA-400; Stressgen; 1:10,000), mouse monoclonal Hsp70 antibody 4G4 (ab5444; Abcam; 1:5,000), and mouse monoclonal Hsp90 antibody (610418; BD Biosciences; 1:1,000). Next, blots were incubated with fluorescent secondary antibodies IRDye® 800CW conjugate goat anti-rabbit IgG and IRDye® 680 conjugated goat anti-mouse IgG (926–32211 and 926–32220 respectively; LI-COR Biosciences) according to the manufacturer’s instructions and scanned using a LI-COR Odyssey infrared scanner.

### RNA isolation and microarray analysis

HEK293 cells were cultured for 24 h in the presence or absence of leucine. Total RNA was isolated using Trizol (Invitrogen) and copied into Cy3-labeled or Cy5-labeled cRNA using the Agilent Low RNA Input Linear Amp Kit PLUS (Agilent), or the reverse for the repeat array. Labeled cRNA samples were hybridized to an Agilent Whole Human Genome Microarray Kit (4 × 44K). The arrays were scanned using an Agilent Microarray Scanner. Image analysis and feature extraction were done with Feature Extraction (version 9.5.1, Agilent). We used an arbitrarily chosen signal cut-off of >50.

### Reverse transcription

One microgram of RNA was treated with DNaseI (Amplification grade; RNase-free; Invitrogen). Subsequently, 5 mM MgCl_2_, RT buffer, 1 mM dNTPs, 18.75 U AMV reverse transcriptase, 20 U RNase inhibitors, and 1.25 μM oligo(dT) were added to a total volume of 20 μl. Reverse transcription was performed for 10 min at 25°C, 60 min at 42°C, and 5 min at 95°C. For QPCR analysis, cDNA was 10-fold diluted.

### Electrophoretic mobility shift assay

T-REx HEK293-pcDNA5 cells were cultured for 24 h in the presence or absence of leucine and subsequently heat shocked for 30 min at 45°C, or cultured for 24 h in the presence or absence of lysine or glutamine. T-REx HEK293-wtHSF1 cells were cultured in the presence of doxycycline and exposed to a heat shock for 30 min at 45°C. Cells were immediately harvested and nuclear extracts were prepared using NE-per nuclear and cytoplasmic reagents (Pierce). From the beginning, protease inhibitors were added to the reagents. Extracts were aliquoted and stored at −80°C. Oligonucleotide probes were end-labeled with ^32^P. The sequences of the NSRU and HSE oligonucleotides used in EMSA are listed in Table 1. After end-labeling, the 5′ overhangs of the 2xNSRU oligonucleotide were filled in with unlabeled dNTPs using DNA polymerase I, large (Klenow) fragment. The EMSA protocol was adapted from Klok et al. (1998) and Thiaville et al. (2008). A mixture containing 5 μg nuclear extract and 3 μg poly dIdC in binding buffer [20 mM HEPES pH 7.9, 100 mM KCl, 1 mM EDTA, 1 mM DTT, 4% (v/v) Ficoll, 1× PhosSTOP (Roche)] was incubated for 20 min on ice. Then 0.01 pmol radiolabeled oligonucleotide was added and the samples were incubated for 20 min at room temperature. For supershifts, 1 μg of antibody was added and again the samples were incubated for 20 min at room temperature. DNA–protein complexes were separated on a pre-run 4% polyacrylamide gel in 0.25× TBE with recirculation of the buffer. The gel was dried and signals were visualized using a PhosphorImager.

### Chromatin immunoprecipitation

T-REx HEK293-dnHSF1, HEK293-HSF1K80Q, or HEK293-pcDNA5 cells were cultured for 24 h in the presence or absence of leucine, with or without doxycycline. Chromatin immunoprecipitation was performed as described in Denissov et al. (2007), except that cells were crosslinked for 15 min with 1% formaldehyde. After quenching with 125 mM glycine, cells were washed twice with ice-cold PBS and resuspended in ice-cold lysis buffer (50 mM HEPES–KOH pH 7.6, 140 mM NaCl, 1 mM EDTA pH 8.0, 1% (v/v) Triton X-100, 0.1% NaDOC, and 1× protease inhibitor cocktail). Antibodies used for ChIP were rabbit polyclonal ATF4 antibody (sc-200; Santa Cruz) and rabbit polyclonal HSF1 antibody (SPA-901; Stressgen). ChIP samples were analyzed by QPCR with the primer sets listed in Table 1.

### Quantitative real-time PCR

Quantitative real-time PCR was performed using the ABI/PRISM 7000 sequence detection system with *Power* SYBR® Green PCR Master mix (Applied Biosystems) using the following amplification protocol: 2 min at 50°C followed by 40 cycles of 15 s at 95°C and 1 min at 60°C. Per reaction, 4 μl of diluted cDNA or ChIP material was used and the DNA was amplified using primers for the sequences of interest, listed in Table 1. Two-tailed Student’s *t* tests were performed to calculate the significance of the data.

### Two-dimensional polyacrylamide gel electrophoresis

For 2D analysis (Bollag et al. 1996), 60 μg protein from nuclear extracts (prepared as described above) in 30 μl was resuspended in 120 μl ureum buffer [9.3 M urea, 0.6 M thio-urea, 0.7 M β-mercaptoethanol, 4% (v/v) Triton X-100 (electrophoresis grade, Sigma)]. Then 0.75 μl IPG buffer was added (pH 4–7, GE Healthcare Life Sciences) and samples were centrifuged for 15 min at 20°C. Isoelectric focusing (IEF) was carried out by putting 125 μl of the sample on a 7-cm IPGphor stripholder and adding a Immobuline Drystrip with a pH gradient of 4 to 7. After covering the Drystrip with cover fluid (GE Healthcare Life Sciences), the strip was passively rehydrated for 12 h using the Ettan IPGphor II (GE Healthcare Life Sciences) at 20°C, with a maximum of 50 μA/strip. IEF was performed at 250 V for 250 V h, 500 V for 500 V h, 1,000 V for 1,000 V h, 5,000 V for 30,000 V h. After IEF, strips were equilibrated for 15 min in 5 ml of equilibration buffer (0.1 M Tris–HCl, pH 6.8, 8 M urea, 30% glycerol, 1% SDS) containing 5 mg/ml DTT, followed by equilibration for 15 min in equilibration buffer containing 45 mg/ml iodoacetamide. Strips were then run on a 12% SDS–polyacrylamide gel, proteins were transferred to nitrocellulose membrane, and western blot analysis was performed.

## Results

### HSF1 loses its DNA binding affinity upon leucine starvation

**Fig. 1:**
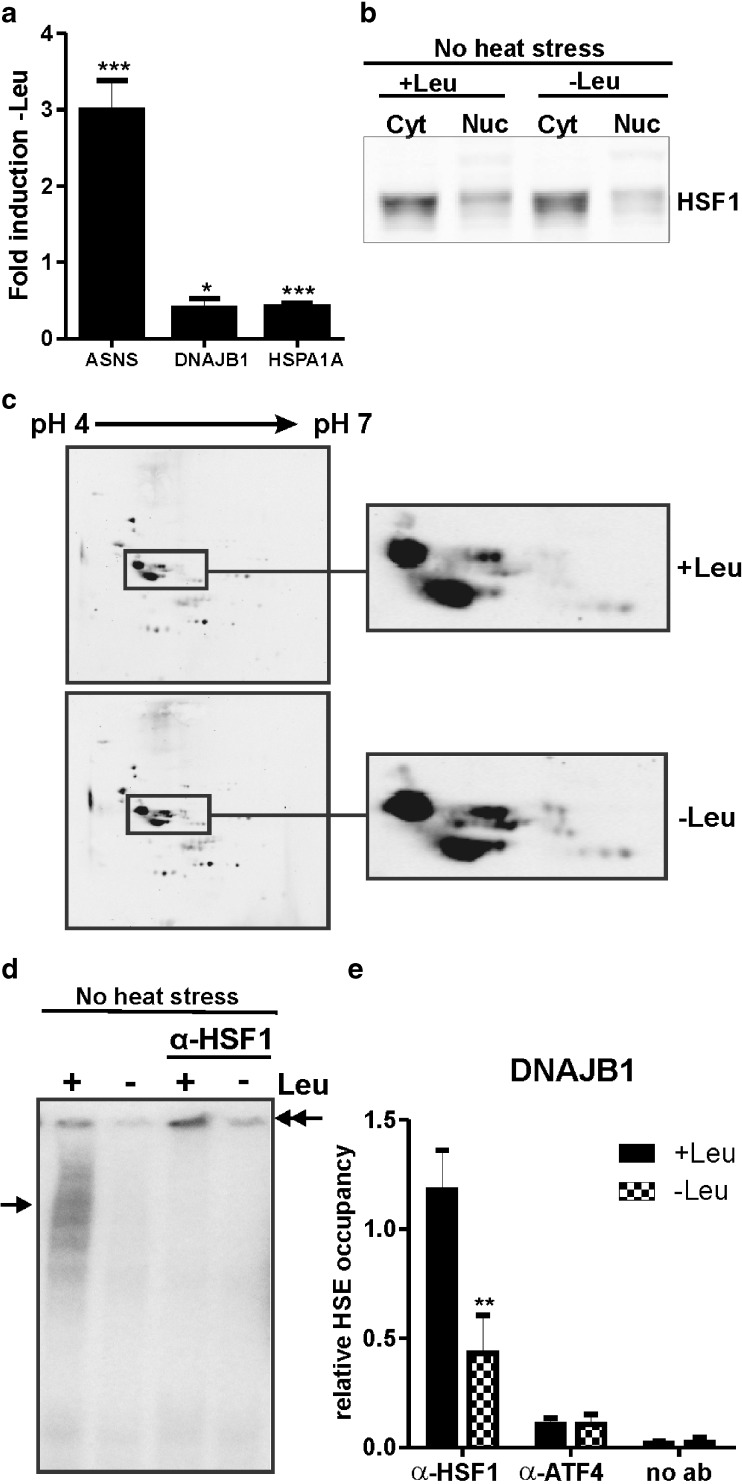
HSF1 loses its DNA binding affinity upon leucine starvation. **a** QPCR validation of ASNS, DNAJB1, and HSPA1A mRNA levels relative to GAPDH mRNA levels upon leucine starvation. *Error bars* represent SD; *P < 0.05; ***P < 0.001, relative to +Leu. **b** HEK293 cells were deprived of leucine for 24 h. Cytoplasmic (*Cyt*) and nuclear (*Nuc*) extracts were made and subjected to SDS–PAGE and western blot analysis using an anti-HSF1 antibody. **c** Nuclear extracts were subjected to 2D gel electrophoresis and western blot analysis using an anti-HSF1 antibody. **d** Nuclear extracts were used in an electrophoretic mobility shift assay with a double-stranded oligonucleotide with the HSE sequence. Supershifts were induced with an anti-HSF1 antibody. *Single arrows* indicate the primary complexes formed; *double arrows* indicate the supershifted complexes. **e** Chromatin immunoprecipitation was performed using an anti-HSF1 or an anti-ATF4 antibody. Bound chromatin was analyzed by QPCR using a primer set surrounding the HSE of the DNAJB1 promoter. As a control, the ChIP was performed without an antibody. *Error bars* represent SD; **P < 0.01, relative to +Leu

A microarray analysis of the transcriptome changes in leucine starved HEK293 cells showed a significant loss of HSPA1A (Hsp70) mRNA, with only 18% left after 24 h of leucine deficiency (Table 2). We noted that the transcript levels of some other HSF1 target genes such as *HSPE1*, *STIP1*, *DNAJB1* (*Hsp40*), and *DNAJA1* were also lower in leucine starved cells, but their decrease was less than 2-fold. The decrease in HSPA1A and DNAJB1 mRNA levels was confirmed by QPCR (Fig. 1a). We have previously shown that in HEK293 cells DNAJB1 mRNA levels rapidly drop when HSF1 is inhibited (Heldens et al. 2010), and these data suggested to us that HSF1 might be inactivated in cells deprived of leucine. We thus looked at HSF1 in leucine starved cells. In extracts of unstressed cells, HSF1 is mostly found in the cytoplasmic fraction and that does not change upon amino acid deprivation (Fig. 1b). HSF1 is known to be extensively modified upon activation, primarily by phosphorylation, which results in altered mobility of HSF1 on 1D and 2D gel electrophoresis. The electrophoretic mobility pattern of nuclear HSF1 did not change in leucine starved cells, indicating that there are no major changes in the modification state of HSF1 (Fig. 1b, c).

**Table 2:** Genes of which the transcript levels were downregulated more than 2-fold upon leucine starvation (24 h)

Gene name	Acc. no.	Description	Fold induction −Leu
HSPA1A	NM_005345	Heat shock 70 kDa protein 1A	0.18
LDLR	NM_000527	Low density lipoprotein receptor (familial hypercholesterolemia)	0.28
SC4MOL	NM_006745	Sterol-C4-methyl oxidase-like	0.38
IFIT1	NM_001548	Interferon-induced protein with tetratricopeptide repeats 1	0.47
INSIG1	NM_198336	Insulin induced gene 1	0.48
IDI1	NM_004508	Isopentenyl-diphosphate delta isomerase 1	0.50
TBX18	ENST00000330469	T-box 18, mRNA (cDNA clone IMAGE:6023106), partial cds.	0.50
EFHB	BC043212	cDNA clone IMAGE:5295205, with apparent retained intron	0.50

We then tested whether the nuclear HSF1 is competent to bind the heat shock element (HSE) using EMSA. HSF1 in nuclear extracts from unstressed cells cultured in the presence of leucine bound the HSE: a clear band shift was seen and the band was supershifted by an antibody to HSF1, indicating that it is indeed HSF1 that is bound (Fig. 1d). However, we could not detect a band shift using nuclear extracts isolated from leucine deprived cells, suggesting that HSF1 is unable to bind to the HSE. As a control, we measured binding of ATF4 and C/EBPβ to the NSRU sequence of the ASNS promoter using these extracts (Fig. S1a). To show that the loss of DNA binding as assayed by EMSA indeed reflects loss of DNA bound HSF1, we performed a ChIP assay with HEK293 cells that were deprived of leucine for 24 h, using a primer set surrounding the HSE of the DNAJB1 promoter. The binding of HSF1 to the DNAJB1 promoter was decreased by about 50% upon leucine starvation (Fig. 1e), whereas ATF4 binding to the ASNS promoter was nicely increased in leucine starved cells (Fig. S1b). This confirms that HSF1 loses its binding affinity upon leucine deprivation.

**Fig. 2:**
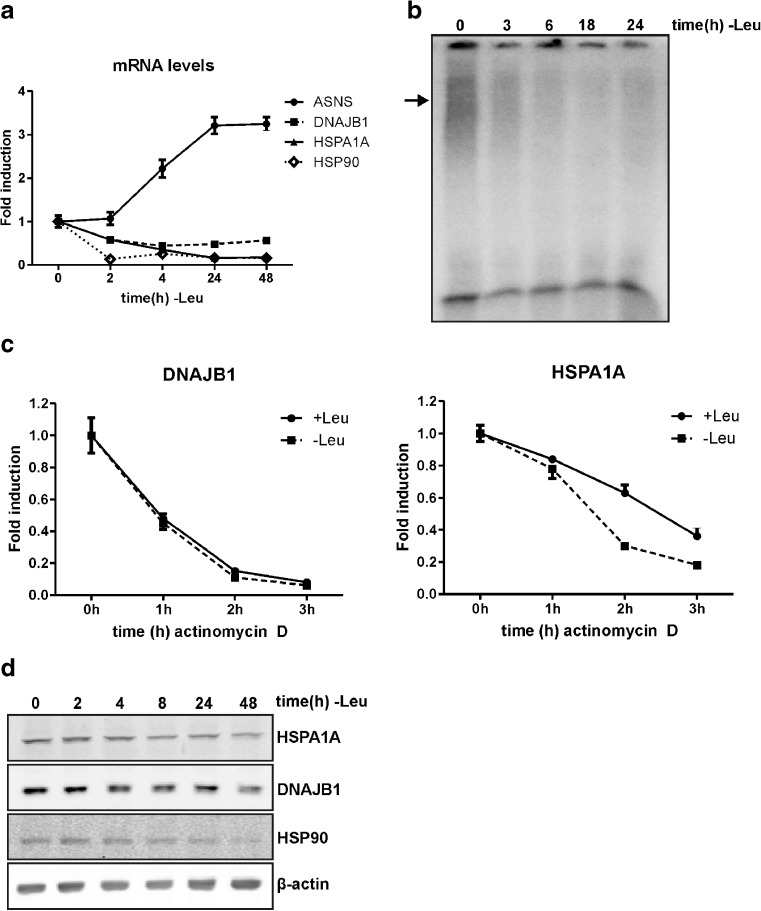
HSP mRNA and HSP protein levels are decreased upon leucine starvation**. a** HEK293 cells were starved for leucine and harvested at the indicated time points. mRNA levels were determined by QPCR analysis and are shown relative to GAPDH mRNA levels. **b** Nuclear extracts were used in an EMSA with a double-stranded oligonucleotide with the HSE sequence. The *arrow* indicates the primary complex formed. **c** HEK293 cells were starved for leucine and after 30 min 5 μg/ml actinomycin D was added to block transcription. Cells were harvested at the indicated time points. mRNA levels were determined by QPCR analysis and are shown relative to GAPDH mRNA levels. **d** Lysates were subjected to SDS–PAGE and western blot analysis using antibodies against the indicated proteins

To determine whether the loss of HSF1 activity is an early or a late event during leucine starvation, we followed the decay of DNAJB1, HSPA1A, and HSP90AA1 mRNA levels with time after leucine starvation. Within 2 h after withdrawal of leucine, these mRNA levels were already strongly decreased (Fig. 2a), while the ASNS mRNA level, indicative of the amino acid response, had not yet risen. In agreement with these findings, the HSF1 binding activity also rapidly decreased after leucine deprivation: less HSF1/HSE complex was detected using nuclear extracts of cells starved for leucine for 3 h (Fig. 2b). Inactivation of HSF1 is thus an early event in the response to lack of leucine. As it has previously been described that glutamine starvation of U937 cells results in loss of Hsp70 through decreased mRNA stability (Eliasen et al. 2006a; Eliasen et al. 2006b), we examined the effect of leucine deprivation on HSPA1A and DNAJB1 mRNA stability in HEK293 cells. Cells were cultured for 30 min in medium with or without leucine, and subsequently actinomycin D was added to block transcription and the level of the transcripts was analyzed. The rate of loss of the DNAJB1 mRNA levels did not differ between unstarved and starved cells (Fig. 2c). However, HSPA1A mRNA levels did show a faster degradation rate in leucine starved cells compared to control cells, indicating decreased mRNA stability. Both the loss in binding activity of HSF1 and decreased stability thus contribute to the lower HSPA1A mRNA levels in leucine starved cells, whereas for DNAJB1 mRNA only the inactivation of HSF1 results in decreased mRNA levels. To see whether reduced heat shock protein mRNA levels also led to a reduction in their corresponding protein levels, we examined HSPA1A, DNAJB1, and HSP90 protein levels upon starvation for leucine. The levels of all three heat shock proteins did decrease upon leucine starvation (Fig. 2d), but not as markedly as the mRNA levels. Presumably these proteins are quite stable.

**Fig. 3:**
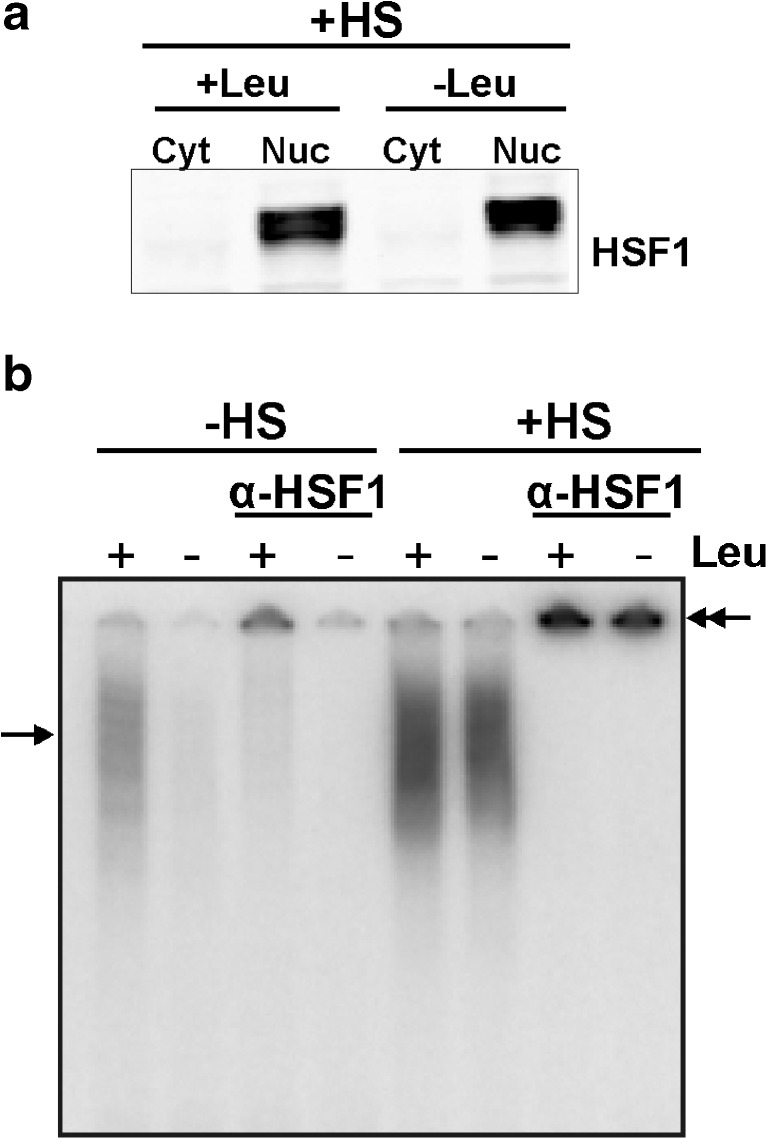
Leucine starved cells can still respond to a proteotoxic insult. **a** HEK293 cells were deprived of leucine for 24 h and exposed to a heat shock for 30 min at 45°C. Cytoplasmic (*Cyt*) and nuclear (*Nuc*) extracts were made and subjected to SDS–PAGE and western blot analysis using an anti-HSF1 antibody to determine HSF1 localization. The results obtained with extracts from unstressed cells isolated in parallel are shown in Fig. 1b. **b** Nuclear extracts of stressed and unstressed cells were used in an electrophoretic mobility shift assay with a double-stranded oligonucleotide with the HSE sequence. Supershifts were induced with an anti-HSF1 antibody. *Single arrows* indicate the primary complexes formed; *double arrows* indicate the supershifted complexes

Leucine starvation thus decreases the endogenous heat shock protein levels, leading to a decreased chaperoning capacity making the cells more sensitive to proteotoxic stress. This raises the question whether leucine starved cells can respond to a proteotoxic insult. To test this, we exposed cells deprived of leucine to a heat stress. The heat shocked leucine deprived cells behaved normally: HSF1 was now found predominantly in the nuclear fraction (Fig. 3a; western blot results obtained with nuclear extracts of unstressed cells are shown in Fig. 1b) and was HSE binding competent (Fig. 3b). Apparently in unstressed cells, it is just the small fraction of active HSF1 that loses DNA binding capacity upon leucine deprivation; the majority of HSF1 is inactive and can still be activated by proteotoxic stress. As HSF1 cycles between the inactive monomeric state and the active trimeric state (Anckar and Sistonen 2011), it is possible that upon longer periods of leucine starvation more HSF1 is inactivated.

### Amino acid starvation in general inactivates HSF1

**Fig. 4:**
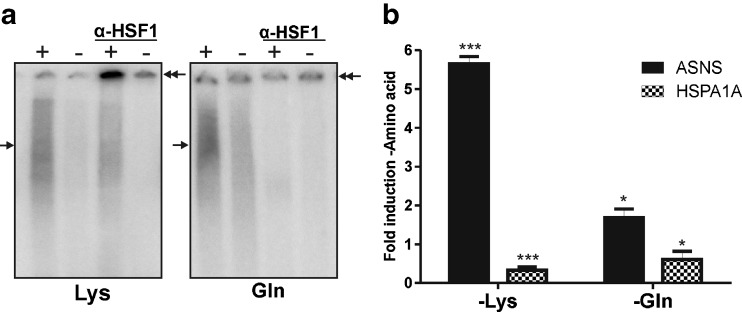
Lysine and glutamine starvation also inactivate nuclear HSF1. **a** HEK293 cells were cultured in the presence of all amino acids (+) or deprived of lysine or glutamine (−) for 24 h. EMSA was performed with a double-stranded oligonucleotide with the HSE sequence. Supershifts were induced with an anti-HSF1 antibody. *Single arrows* indicate the primary complexes formed; *double arrows* indicate the supershifted complexes. **b** ASNS and HSPA1A mRNA levels were determined by QPCR analysis and are shown relative to GAPDH mRNA levels. *Error bars* represent SD; *P < 0.05; ***P < 0.001, relative to +amino acid

The amino acid response is a response induced by lack of amino acids, and if inactivation of HSF1 is part of the general amino acid response, then the HSF1 DNA binding affinity should also be affected upon starvation for other amino acids. We therefore used EMSA to examine the effects on HSF1 binding upon starvation for two other amino acids: lysine and glutamine. The HSF1/HSE band shift that was detected using nuclear extracts from non-starved cells strongly decreased upon starvation for either of the amino acids (Fig. 4a), indicating that amino acid starvation in general leads to inactivation of HSF1.

Next, we tested the effect of starvation for lysine and glutamine on HSPA1A mRNA levels. ASNS mRNA levels were analyzed as a control for induction of the amino acid response: starvation for either of the two amino acids induced ASNS mRNA levels (Fig. 4b). HSPA1A mRNA levels were decreased upon starvation for both lysine and glutamine, suggesting a general effect of amino acid starvation on heat shock protein mRNA levels, where, at least in the case of HSPA1A mRNA, decreased mRNA stability also plays a role.

### Amino acid limitation also affects HSF1 binding affinity and HSP mRNA levels

**Fig. 5:**
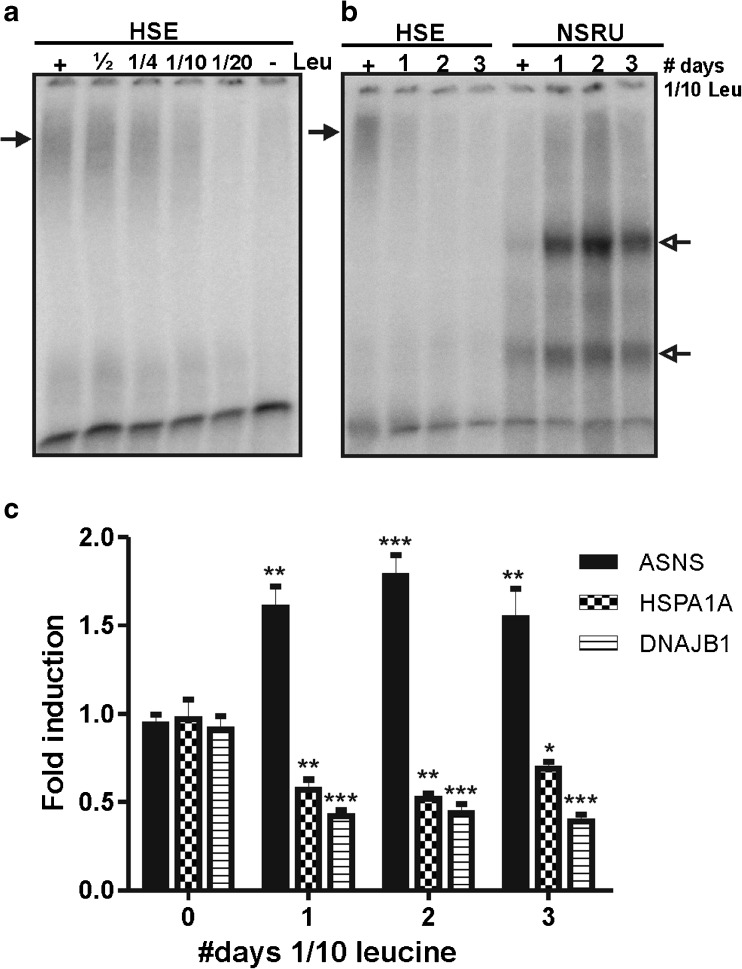
Amino acid limitation affects HSF1 binding affinity and HSP mRNA levels. **a** HEK293 cells were cultured in medium containing limiting amounts of leucine for 24 h. The concentrations are indicated relative to the standard leucine concentration in medium (+), which is 450 μM. EMSA was performed with a double-stranded oligonucleotide with the HSE sequence. The *arrow* indicates the primary complexes formed. **b** HEK293 cells were cultured for the indicated times in medium containing 45 μM leucine. As a control, cells were cultured in parallel in standard medium containing 450 μM leucine (+). Medium was changed every day. Nuclear extracts were used in EMSA with a double-stranded oligonucleotide with the HSE or NSRU sequence. The *closed arrow* indicates the HSE complex formed. *Open arrows* indicate the NSRU complexes formed. **c** ASNS, DNAJB1, and HSPA1A mRNA levels were determined by QPCR analysis and are shown relative to GAPDH mRNA levels. *Error bars* represent SD; *P < 0.05; **P < 0.01; ***P < 0.001, relative to 0 days

All experiments reported above were performed with medium completely lacking leucine, lysine, or glutamine—an extreme situation. A more physiological situation would be a shortage but not a complete lack of an amino acid. To test whether the HSF1 binding affinity is also lost under conditions of amino acid limitation, we analyzed the binding of HSF1 to a HSE by EMSA using nuclear extracts of cells that were cultured in medium with decreasing leucine concentrations. At one tenth of the normal concentration of leucine in the medium, a loss in HSF1/HSE binding was found (Fig. 5a); at this concentration, cells were still slowly growing. Culturing the cells for several days in medium containing one tenth of the normal leucine concentration led to a further decrease in HSF1 binding; at the same time, NSRU–protein complexes became detectable (Fig. 5b).

We then determined the effect of culturing the cells in medium with one tenth of the normal leucine concentration on heat shock protein mRNA levels. Already after 1 day, a decrease in HSPA1A and DNAJB1 mRNA levels was detected (Fig. 5c), and this decrease persisted after 2 and 3 days of culturing in medium with limiting leucine concentrations. The ASNS mRNA level increased upon leucine limitation. These results indicate that amino acid limitation also affects at least the HSPA1A and DNAJB1 mRNA levels. We were unable to detect a decrease in HSPA1A and DNAJB1 protein levels upon leucine limitation for 3 days (Fig. S2). This was not quite unexpected, as we only found a small effect on protein levels after complete leucine starvation (Fig. 2c). Unfortunately, it is experimentally not possible to test cells cultured for a longer time: passaging cells induces a transient heat shock response.

### HSF1 can bind to the NSRU of the ASNS promoter

**Fig. 6:**
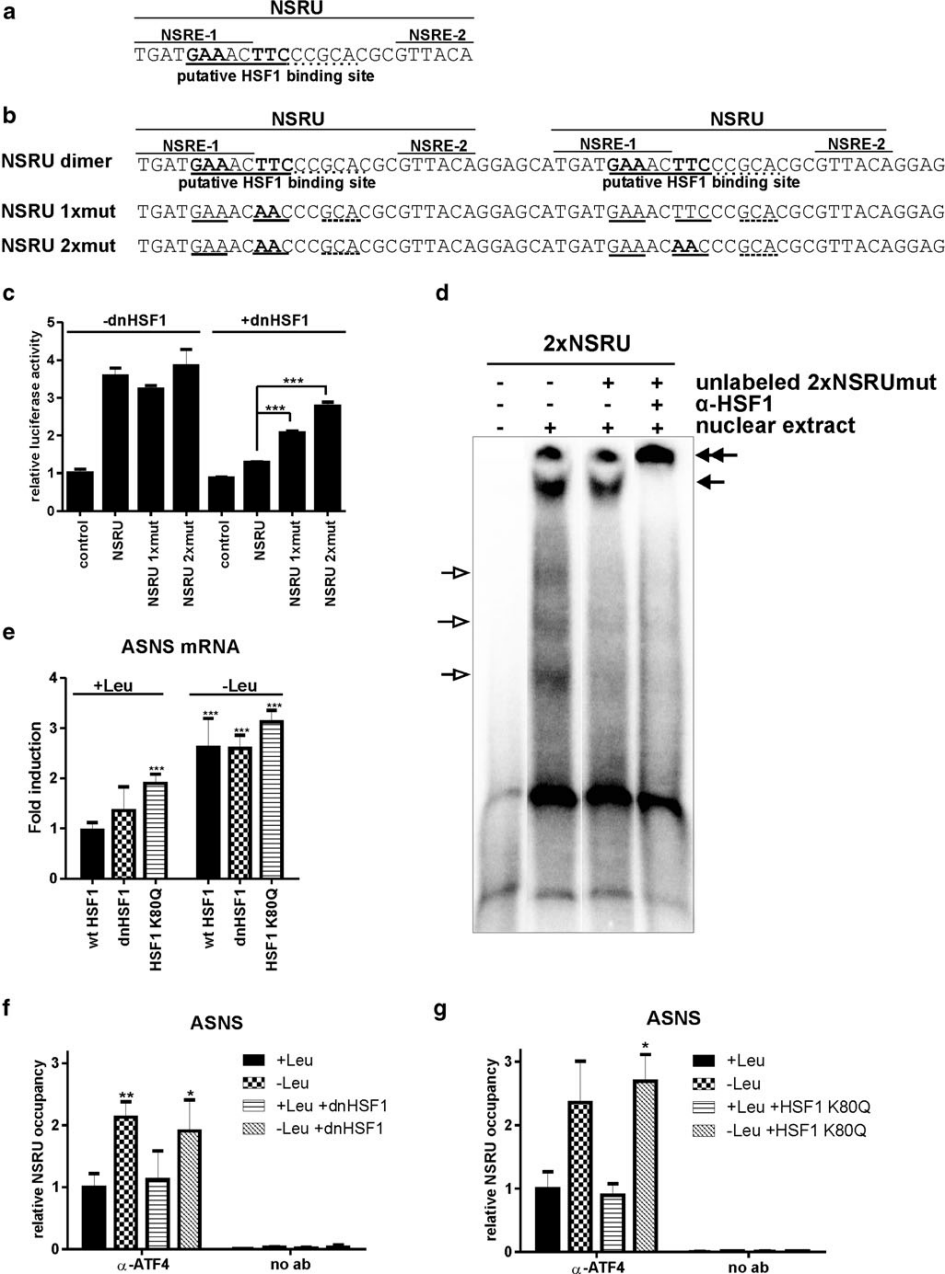
The ASNS NSRU contains a HSE. **a** Sequence of the NSRU of the ASNS promoter. The putative HSF1 binding sequence is *underlined*. **b** Sequence of a tandem repeat of the nutrient sensing response unit of the ASNS promoter used in the reporter plasmid. The putative HSF1 binding sequence is *underlined*. NSRU 1xmut and NSRU 2xmut are the sequences that are mutated for the putative HSE (indicated in *bold*). **c** HEK-dnHSF1 cells were transfected with the indicated NSRU reporter plasmid and treated with doxycycline. Cells were harvested and assayed for reporter gene activities. The results shown are the average of three independent transfections. **d** HEK-wtHSF1 cells were cultured in the presence of doxycyline and heat stressed for 30 min at 45°C. Directly after heat stress, nuclear extracts were made. EMSA was performed with a double-stranded oligonucleotide with the 2xNSRU sequence. Where indicated, a 2-fold molar excess of unlabeled double-stranded oligonucleotide with the 2xNSRUmut sequence was added. Supershifts were induced with an anti-HSF1 antibody. *Open arrows* indicate the ATF4 complexes; *closed arrows* indicate the HSF1 specific complex and the supershifted complex. **e** Doxycycline treated HEK-wtHSF1, HEK-HSF1 K80Q, and HEK-dnHSF1 cells were starved for leucine. mRNA levels were determined by QPCR analysis and are shown relative to GAPDH mRNA levels. **f**, **g** Chromatin immunoprecipitation was performed using an ATF4 antibody. Bound chromatin was analyzed by QPCR using a primer set surrounding the NSRU of the ASNS promoter. In all figures, *error bars* represent SD; *P < 0.05; **P < 0.01; ***P < 0.001

The results presented above raise the obvious question as to what the role of HSF1 is in the amino acid response: why is it inactivated as part of this response? To answer this question, we looked at the NSRU of the ASNS promoter, a canonical amino acid response element, and noted a putative HSF1 binding site (Fig. 6a). To test whether this HSE is functional, we inserted a dimer of the NSRU sequence (Fig. 6b) in the pGL3 promoter vector which contains a SV40 promoter-driven luciferase gene. The activity of this NSRU–luc construct was inhibited by a dominant negative HSF1, an HSF1 mutant lacking the activation domain (Heldens et al. 2010; Fig. 6c). Mutation of one of the putative HSEs (NSRU 1xmut; Fig. 6b) increased activity of the NSRU reporter in dnHSF1 expressing cells to about half of that in control cells; mutation of both sites (NSRU 2xmut; Fig. 6b) restored about 75% of the activity. In the absence of dnHSF1 expression, these mutations had no effect (Fig. 6c; −dnHSF1). The HSF1 binding to the putative NSRU HSE is weak: we were unable to detect in vitro binding (EMSA) using a probe containing a single copy of the NSRU. However, when we used a probe containing the NSRU repeat, as present in the NSRU luciferase reporter construct, and nuclear extracts of heat stressed cells overexpressing wtHSF1, complexes could be detected (Fig. 6d). The signals of the faster migrating complexes (indicated by open arrows) decreased when unlabeled oligonucleotides with the mutated HSF1 binding sites (NSRU 2xmut; Fig. 6d) were used and likely represent ATF4 complexes. The slowly migrating complex (indicated by a single closed arrow) could not be competed for by the NSRU 2xmut and was supershifted by an HSF1 antibody (indicated by double arrows). This complex thus represents HSF1 binding. These data demonstrate that HSF1 can indeed bind to the NSRU sequence of the ASNS promoter.

In vivo, the NSRU is part of a larger promoter region and the activity of a NSRU–luc construct does not necessarily reflect that of the ASNS promoter. In leucine fed cells expressing the dnHSF1 mutant, the ASNS transcript level did not decrease but rather increased slightly while the level in leucine starved cells was not affected (Fig. 6e). When we used cells expressing an HSF1 mutant unable to bind DNA [(HSF1 K80Q (Westerheide et al. 2009), this mutant also blocks the heat shock induction of HSF1 target genes (Fig. S3)] the ASNS mRNA level increased slightly in both leucine fed and leucine starved cells (Fig. 6e). We were also unable to detect binding of HSF1 to the NSRU in vivo by ChIP. Finally, expression of dnHSF1 or HSF1 K80Q did not influence the extent of binding of ATF4, the main activating transcription factor bound to the NSRU, either in the presence or absence of leucine (Fig. 6f, g).

### Effect of dnHSF1 or HSF1 K80Q on the transcript levels of amino acid response genes

The results presented above show that even though the NSRU of the ASNS promoter contains an HSE, HSF1 does not appear to regulate expression of the ASNS gene. We therefore looked whether the transcript levels of other amino acid responsive genes are affected in cells expressing HSF1 K80Q or dnHSF1. We identified the amino acid responsive genes in HEK293 cells starved for leucine by microarray analysis (Table S1) and then looked at the change in transcript level of these genes when either HSF1 K80Q or dnHSF1 was expressed. As seen in Fig. S4 and Table 3, in dnHSF1 expressing cells on average the transcript levels increased. A more varied response was seen in HSF1 K80Q cells with an increase in some and a decrease in others. We noted that there appears to be a large cell and even amino acid specific effect to the amino acid deprivation response: a comparison of our results with published microarray studies (Deval et al. 2009; Sikalidis et al. 2011; Shan et al. 2010) showed that the transcript levels of only six genes changed significantly in all cases. Five of these encode transcription factors (ATF3, CEBPB, CEBPG, KLF10, TRIB3) and are all upregulated; one encodes the enzyme ASNS. The transcript level of these canonical amino acid response genes also increased in leucine fed dnHSF1 expressing HEK293 cells; in HSF1 K80Q expressing HEK293 cells, only a subset showed an increase (Table 3).

**Table 3:** Changes in transcript levels of “canonical” amino acid responsive genes upon expression of dnHSF1 or HSF1 K80Q

Fold change				
Gene name	Acc. no.	Description	dnHSF1	HSF1 K80Q
ASNS	NM_001673	Asparagine synthetase	1.71	1.99
ATF3	NM_004024	Activating transcription factor 3	1.18	0.83
CEBPB	NM_005194	CCAAT/enhancer binding protein (C/EBP), beta	1.54	1.18
CEBPG	NM_001806	CCAAT/enhancer binding protein (C/EBP), gamma	1.29	1.26
DDIT3	NM_004083	DNA-damage-inducible transcript 3 (CHOP)	1.43	1.11
KLF10	NM_005655	Kruppel-like factor 10	1.20	0.74
TRIB3	NM_021158	Tribbles homolog 3 (*Drosophila*)	1.30	0.93

Most, if not all, of the canonical amino acid responsive genes are direct targets of ATF4, and the increase in their transcript level in leucine fed dnHSF1 expressing cells could thus be due to an increase in ATF4 levels. However, we could not detect a significant effect of dnHSF1 on the expression levels of ATF4 or C/EBPβ, another transcription factor involved in the amino acid response, either in the presence or absence of leucine (Fig. S5).

## Discussion

We have shown here that, unusually and unexpectedly, HSF1 is not activated but silenced when cells are starved for amino acids. In leucine, glutamine, or lysine starved cells, HSF1 in the nuclear fraction lost its DNA binding activity. HSF1 activity is regulated via complex regulatory mechanisms, including post-translational modifications. HSF1 can for example be regulated by phosphorylation (Guettouche et al. 2005; for review, see also Holmberg et al. 2002). However, we do not see a change in electrophoretic mobility, making extensive changes in the phosphorylation pattern unlikely. It has also been described that acetylation of HSF1 at K80 results in a loss in DNA binding affinity (Westerheide et al. 2009); a distinct possibility is thus that upon amino acid deprivation HSF1 is acetylated at K80. We could not show acetylation of HSF1 using an antibody directed against acetylated lysine, but this could well have been an experimental problem.

A possible reason for the inactivation of HSF1 in amino acid starved cells is that HSF1 is directly involved in regulating the activity of amino acid starvation responsive genes in fed cells, as suggested by the finding of an HSF1 binding site in the NSRU of the ASNS promoter (Fig. 6a), as well as in other amino acid response promoters such as those of the ATF3 (Takii et al. 2010) and S100P (unpublished data) genes. However, we could not find direct evidence for HSF1 binding to the NSRU in vivo. Furthermore, the effect of exogenous expression of either a non-DNA binding or a dominant negative mutant of HSF1 on the transcript levels of amino acid starvation responsive genes is not large, while depleting the cells of HSF1 by siRNA had no effect on at least the level of ASNS mRNA (data not shown). Hence, although it is tempting to suggest that HSF1 is a regulatory factor in the amino acid starvation response, we could find no solid evidence that that is indeed the case.

HSF1 is best known for its activation of transcription of the heat shock protein genes during proteotoxic stress, like heat, UV, and viral infections, but HSF1 also plays a physiological role in setting the circadian rhythm (Reinke et al. 2008). For example, the circadian clock gene Per2 is an HSF1 target (Kornmann et al. 2007; Tamaru et al. 2011). Intriguingly, one of the genes at the core of the amino acid deprivation response, *KLF10*, is also a target gene of a clock protein (Guillaumond et al. 2010). Perhaps the inactivation of HSF1 during the amino acid response is part of the intricate crosstalk between metabolism and the circadian rhythm (for review, see Asher and Schibler 2011).

Alternatively, inactivation of HSF1 during a non-proteotoxic stress response may be a more general phenomenon and may either prevent diversion of cellular resources to dealing with a secondary problem or aid the organism in clearing irreversibly damaged cells. Recently, it was shown that HSF1 is also inactivated during the DNA damage response (Kim et al. 2012), an inactivation that facilitates senescence.

We do see the loss of HSF1 binding and the change in HSPA1A stability during amino acid starvation reflected in the levels of mRNAs of HSF1 target genes: the level of both HSPA1A (Hsp70) and DNAJB1 (Hsp40) mRNAs drops markedly (Fig. 1a). A significant decrease in DNAJB1 mRNA was also noted in leucine starved MEFs (Deval et al. 2009), while HSPA1A mRNA was reported to decrease significantly in cysteine and histidine starved HepG2 cells (Sikalidis et al. 2011; Shan et al. 2010). In *Drosophila* larvae, complete starvation or sugar deprivation led to a strong decrease in Hsp90 mRNA levels (Zinke et al. 2002). The loss of HSF1 binding affinity and the decrease in HSPA1A and DNAJB1 mRNA levels was not just seen in cells dying from lack of an amino acid but also in cells fed just enough leucine to continue slow growth (Fig. 5). In the future, it needs to be tested whether whole organisms show the same response when amino acids are limiting. If so, then malnutrition, lack of essential amino acids, would also lead to cellular (and organismal) frailty due to a loss of chaperoning capacity.

## Electronic supplementary material

Below is the link to the electronic supplementary material.

ESM 1 (DOCX 277 kb)

## Abbreviations

GCN2: General control non-derespressible
ATF: Activating transcription factor
AARE: Amino acid response element
NSRU: Nutrient sensing response unit
ASNS: Asparagine synthetase
CHOP: C/EBP homologous protein
C/EBP: CCAAT/enhancer binding protein
UPR: Unfolded protein response
PERK: Double-stranded RNA-activated protein kinase-like ER kinase
PKR: Double-stranded RNA-activated protein kinase
HRI: Heme-regulated inhibitor
HSF1: Heat shock factor 1
HSE: Heat shock element
MEF: Mouse embryonic fibroblast

## References

[CR1] Anckar J, Sistonen L (2011). Regulation of HSF1 function in the heat stress response: implications in aging and disease. Annu Rev Biochem.

[CR2] Asher G, Schibler U (2011). Crosstalk between components of circadian and metabolic cycles in mammals. Cell Metab.

[CR3] Averous J, Bruhat A, Jousse C, Carraro V, Thiel G, Fafournoux P (2004). Induction of CHOP expression by amino acid limitation requires both ATF4 expression and ATF2 phosphorylation. J Biol Chem.

[CR4] Bollag DM, Rozycki MD, Edelstein SJ (1996). Protein methods.

[CR5] Bruhat A, Cherasse Y, Maurin AC, Breitwieser W, Parry L, Deval C, Jones N, Jousse C, Fafournoux P (2007). ATF2 is required for amino acid-regulated transcription by orchestrating specific histone acetylation. Nucleic Acids Res.

[CR6] Bruhat A, Jousse C, Carraro V, Reimold AM, Ferrara M, Fafournoux P (2000). Amino acids control mammalian gene transcription: activating transcription factor 2 is essential for the amino acid responsiveness of the CHOP promoter. Mol Cell Biol.

[CR7] Chen H, Pan YX, Dudenhausen EE, Kilberg MS (2004). Amino acid deprivation induces the transcription rate of the human asparagine synthetase gene through a timed program of expression and promoter binding of nutrient-responsive basic region/leucine zipper transcription factors as well as localized histone acetylation. J Biol Chem.

[CR8] Denissov S, van Driel M, Voit R, Hekkelman M, Hulsen T, Hernandez N, Grummt I, Wehrens R, Stunnenberg H (2007). Identification of novel functional TBP-binding sites and general factor repertoires. EMBO J.

[CR9] Deval C, Chaveroux C, Maurin AC, Cherasse Y, Parry L, Carraro V, Milenkovic D, Ferrara M, Bruhat A, Jousse C, Fafournoux P (2009). Amino acid limitation regulates the expression of genes involved in several specific biological processes through GCN2-dependent and GCN2-independent pathways. FEBS J.

[CR10] Eliasen MM, Brabec M, Gerner C, Pollheimer J, Auer H, Zellner M, Weingartmann G, Garo F, Roth E, Oehler R (2006). Reduced stress tolerance of glutamine-deprived human monocytic cells is associated with selective down-regulation of Hsp70 by decreased mRNA stability. J Mol Med.

[CR11] Eliasen MM, Winkler W, Jordan V, Pokar M, Marchetti M, Roth E, Allmaier G, Oehler R (2006). Adaptive cellular mechanisms in response to glutamine-starvation. Front Biosci.

[CR12] Gjymishka A, Palii SS, Shan J, Kilberg MS (2008). Despite increased ATF4 binding at the C/EBP-ATF composite site following activation of the unfolded protein response, system A transporter 2 (SNAT2) transcription activity is repressed in HepG2 cells. J Biol Chem.

[CR13] Guettouche T, Boellmann F, Lane WS, Voellmy R (2005). Analysis of phosphorylation of human heat shock factor 1 in cells experiencing a stress. BMC Biochem.

[CR14] Guillaumond F, Grechez-Cassiau A, Subramaniam M, Brangolo S, Peteri-Brunback B, Staels B, Fievet C, Spelsberg TC, Delaunay F, Teboul M (2010). Kruppel-like factor KLF10 is a link between the circadian clock and metabolism in liver. Mol Cell Biol.

[CR15] Harding HP, Novoa I, Zhang Y, Zeng H, Wek R, Schapira M, Ron D (2000). Regulated translation initiation controls stress-induced gene expression in mammalian cells. Mol Cell.

[CR16] Heldens L, Dirks RP, Hensen SM, Onnekink C, van Genesen ST, Rustenburg F, Lubsen NH (2010). Co-chaperones are limiting in a depleted chaperone network. Cell Mol Life Sci.

[CR17] Holcik M, Sonenberg N (2005). Translational control in stress and apoptosis. Nat Rev Mol Cell Biol.

[CR18] Holmberg CI, Tran SE, Eriksson JE, Sistonen L (2002). Multisite phosphorylation provides sophisticated regulation of transcription factors. Trends Biochem Sci.

[CR19] Kim G, Meriin AB, Gabai VL, Christians E, Benjamin I, Wilson A, Wolozin B, Sherman MY (2012) The heat shock transcription factor Hsf1 is downregulated in DNA damage-associated senescence, contributing to the maintenance of senescence phenotype. Aging Cell. doi:10.1111/j.1474-9726.2012.00827.x 10.1111/j.1474-9726.2012.00827.x PMC3433748 22510478

[CR20] Klok EJ, van Genesen ST, Civil A, Schoenmakers JG, Lubsen NH (1998). Regulation of expression within a gene family. The case of the rat gammaB- and gammaD-crystallin promoters. J Biol Chem.

[CR21] Kornmann B, Schaad O, Bujard H, Takahashi JS, Schibler U (2007). System-driven and oscillator-dependent circadian transcription in mice with a conditionally active liver clock. PLoS Biol.

[CR22] Krishnamoorthy T, Pavitt GD, Zhang F, Dever TE, Hinnebusch AG (2001). Tight binding of the phosphorylated alpha subunit of initiation factor 2 (eIF2alpha) to the regulatory subunits of guanine nucleotide exchange factor eIF2B is required for inhibition of translation initiation. Mol Cell Biol.

[CR23] Morimoto RI (1998). Regulation of the heat shock transcriptional response: cross talk between a family of heat shock factors, molecular chaperones, and negative regulators. Genes Dev.

[CR24] Page TJ, Sikder D, Yang L, Pluta L, Wolfinger RD, Kodadek T, Thomas RS (2006). Genome-wide analysis of human HSF1 signaling reveals a transcriptional program linked to cellular adaptation and survival. Mol Biosyst.

[CR25] Reinke H, Saini C, Fleury-Olela F, Dibner C, Benjamin IJ, Schibler U (2008). Differential display of DNA-binding proteins reveals heat-shock factor 1 as a circadian transcription factor. Genes Dev.

[CR26] Shan J, Lopez MC, Baker HV, Kilberg MS (2010). Expression profiling after activation of the amino acid deprivation response in HepG2 human hepatoma cells. Physiol Genomics.

[CR27] Sikalidis AK, Lee JI, Stipanuk MH (2011). Gene expression and integrated stress response in HepG2/C3A cells cultured in amino acid deficient medium. Amino Acids.

[CR28] Siu F, Bain PJ, LeBlanc-Chaffin R, Chen H, Kilberg MS (2002). ATF4 is a mediator of the nutrient-sensing response pathway that activates the human asparagine synthetase gene. J Biol Chem.

[CR29] Takii R, Inouye S, Fujimoto M, Nakamura T, Shinkawa T, Prakasam R, Tan K, Hayashida N, Ichikawa H, Hai T, Nakai A (2010). Heat shock transcription factor 1 inhibits expression of IL-6 through activating transcription factor 3. J Immunol.

[CR30] Tamaru T, Hattori M, Honda K, Benjamin I, Ozawa T, Takamatsu K (2011). Synchronization of circadian Per2 rhythms and HSF1-BMAL1:CLOCK interaction in mouse fibroblasts after short-term heat shock pulse. PLoS One.

[CR31] Thiaville MM, Dudenhausen EE, Zhong C, Pan YX, Kilberg MS (2008). Deprivation of protein or amino acid induces C/EBPbeta synthesis and binding to amino acid response elements, but its action is not an absolute requirement for enhanced transcription. Biochem J.

[CR32] Voellmy R (2004). On mechanisms that control heat shock transcription factor activity in metazoan cells. Cell Stress Chaperones.

[CR33] Wek RC, Jiang HY, Anthony TG (2006). Coping with stress: eIF2 kinases and translational control. Biochem Soc Trans.

[CR34] Westerheide SD, Anckar J, Stevens SM, Sistonen L, Morimoto RI (2009). Stress-inducible regulation of heat shock factor 1 by the deacetylase SIRT1. Science.

[CR35] Wu C (1995). Heat shock transcription factors: structure and regulation. Annu Rev Cell Dev Biol.

[CR36] Xie Y, Chen C, Stevenson MA, Auron PE, Calderwood SK (2002). Heat shock factor 1 represses transcription of the IL-1beta gene through physical interaction with the nuclear factor of interleukin 6. J Biol Chem.

[CR37] Zhong C, Chen C, Kilberg MS (2003). Characterization of the nutrient-sensing response unit in the human asparagine synthetase promoter. Biochem J.

[CR38] Zinke I, Schutz CS, Katzenberger JD, Bauer M, Pankratz MJ (2002). Nutrient control of gene expression in *Drosophila*: microarray analysis of starvation and sugar-dependent response. EMBO J.

