# Supplementary material for: Heat shock factor 1 is inactivated by amino acid deprivation
Source: Cell Stress Chaperones. 2012 Jul 14;17(6):743–55. doi: 10.1007/s12192-012-0347-1 (PMC3468675; doi:10.1007/s12192-012-0347-1)
Supplement: Supplementary file 1 — (DOCX 277 kb) [file 12192_2012_347_MOESM1_ESM.docx]

**Supplemental data**

**Heat shock factor 1 is inactivated by amino acid deprivation**

*Cell Stress and Chaperones*

**Sanne M.M. Hensen, Lonneke Heldens, Chrissy M.W. van Enckevort, Siebe T. van Genesen, Ger J.M. Pruijn, Nicolette H. Lubsen**

Department of Biomolecular Chemistry, Radboud University Nijmegen, P.O. Box 9101, NL-6500 HB Nijmegen, The Netherlands

Corresponding author: Nicolette H. Lubsen, 271 Department of Biomolecular Chemistry, Radboud University Nijmegen, P.O. Box 9101, NL-6500 HB Nijmegen, The Netherlands.

Telephone: +31-(0)24-3616850. Fax: +31-(0)24-3540525. e-mail address: N.Lubsen@science.ru.nl

**Table S1** Transcript levels induced more than 2-fold upon leucine starvation (24 h)

| **Gene name** | **Acc. No.** | **Description** | **Fold induction**  **-Leu** |
| --- | --- | --- | --- |
| FUT1 | NM_000148 | Fucosyltransferase 1 (galactoside 2-alpha-L-fucosyltransferase, H blood group) | 5.33 |
| SLC7A11 | NM_014331 | Solute carrier family 7, (cationic amino acid transporter, y+ system) member 11 | 5.31 |
| TRIB3 | NM_021158 | Tribbles homolog 3 (Drosophila) | 5.01 |
| NUPR1 | NM_012385 | Nuclear protein 1 | 4.69 |
| DDIT3 | NM_004083 | DNA-damage-inducible transcript 3 | 4.12 |
| ALDH1L2 | CR749561 | mRNA; cDNA DKFZp686A16126 | 3.99 |
| PCK2 | NM_004563 | Phosphoenolpyruvate carboxykinase 2 (mitochondrial) | 3.83 |
| LOC645733 | XM_374004 | Hypothetical LOC389025 | 3.44 |
| TXNIP | NM_006472 | Thioredoxin interacting protein | 3.44 |
| JDP2 | NM_130469 | Jun dimerization protein 2 | 3.43 |
| TTC18 | NM_145170 | Ttetratricopeptide repeat domain 18 | 3.25 |
| GDAP1L1 | NM_024034 | Ganglioside-induced differentiation-associated protein 1-like 1 | 3.24 |
| S100P | NM_005980 | S100 calcium binding protein P | 3.23 |
| CEBPB | NM_005194 | CCAAT/enhancer binding protein beta | 3.22 |
| GDF15 | NM_004864 | Growth differentiation factor 15 | 3.22 |
| ASNSL1 | THC2363646 | AJHYNG asparagine synthase (glutamine-hydrolysing) [similarity] - golden hamster | 3.10 |
| TAC1 | NM_003182 | Tachykinin, precursor 1 | 3.06 |
| ASNS | BC030024 | Asparagine synthetase | 2.89 |
| OGT | NM_181672 | O-linked N-acetylglucosamine (GlcNAc) transferase | 2.85 |
| TTC25 | NM_031421 | Tetratricopeptide repeat domain 25 | 2.84 |
| TIGA1 | NM_053000 | TIGA1 | 2.79 |
| A2LD1 | BC001077 | A2LD1 AIG2-like domain 1 | 2.79 |
| LOC285908 | NM_181722 | NCRNA00174 non-protein coding RNA 174 | 2.77 |
| LOC729779 | LOC729779 | Similar to phosphoserine aminotransferase | 2.68 |
| RAB39B | NM_171998 | RAB39B, member RAS oncogene family | 2.60 |
| ATF3 | NM_004024 | Activating transcription factor 3 | 2.59 |
| RAPGEF3 | NM_006105 | Rap guanine nucleotide exchange factor (GEF) 3 | 2.57 |
| ARRDC4 | NM_183376 | Arrestin domain containing 4 | 2.54 |
| SH3BGR | NM_007341 | SH3 domain binding glutamic acid-rich protein | 2.47 |
| PLEKHG4 | NM_015432 | Pleckstrin homology domain containing, family G (with RhoGef domain) member 4 | 2.47 |
| CALCB | NM_000728 | Calcitonin-related polypeptide, beta | 2.46 |
| AREG | NM_001657 | Amphiregulin (schwannoma-derived growth factor) | 2.43 |
| LOC100130042 | LOC100130042 | Similar to methylenetetrahydrofolate dehydrogenase 2 | 2.43 |
| FCGBP | NM_003890 | Fc fragment of IgG binding protein | 2.40 |
| INHBA | NM_002192 | Inhibin, beta A (activin A, activin AB alpha polypeptide) | 2.40 |
| CCDC11 | NM_145020 | Coiled-coil domain containing 11 | 2.40 |
| LRRIQ1 | NM_032165 | Leucine-rich repeats and IQ motif containing 1 | 2.39 |
| CEBPG | NM_001806 | CCAAT/enhancer binding protein gamma | 2.39 |
| POPDC2 | NM_022135 | Popeye domain containing 2 | 2.37 |
| FLJ31659 | NM_153027 | TRIM61 tripartite motif-containing 61 | 2.35 |
| C1orf24 | NM_052966 | Chromosome 1 open reading frame 24 | 2.35 |
| ENST00000338358 | ENST00000338358 | Hypothetical LOC100130691 | 2.34 |
| KIAA1407 | NM_020817 | KIAA1407, unknown function | 2.34 |
| CR623273 | CR623273 | METTL12 methyltransferase like 12 | 2.34 |
| FBXO4 | NM_012176 | F-box protein 4 | 2.34 |
| LOC51315 | NM_016618 | KRCC1 lysine-rich coiled-coil 1 | 2.32 |
| C21orf69 | NM_058189 | Chromosome 21 open reading frame 69 | 2.29 |
| KLF10 | NM_005655 | Kruppel-like factor 10 | 2.29 |
| C9orf103 | NM_001001551 | Chromosome 9 open reading frame 103 | 2.28 |
| AMT | NM_000481 | Aminomethyltransferase | 2.28 |
| ZNF33B | NM_006955 | Zinc finger protein 33B | 2.26 |
| FLJ37035 | AK094354 | cDNA FLJ37035 fis, clone BRACE2011545 | 2.25 |
| C6orf26 | NM_001039651 | Chromosome 6 open reading frame 26 | 2.24 |
| RBKS | NM_022128 | Ribokinase | 2.23 |
| LPXN | NM_004811 | Leupaxin | 2.22 |
| ELAC1 | NM_018696 | ElaC homolog 1 (E. coli) | 2.22 |
| IL23A | NM_016584 | Interleukin 23, alpha subunit p19 | 2.21 |
| DUSP26 | NM_024025 | Dual specificity phosphatase 26 (putative) | 2.21 |
| FLJ10916 | NM_018271 | THNSL2 threonine synthase-like 2 (S. cerevisiae) | 2.20 |
| CHGB | NM_001819 | Chromogranin B (secretogranin 1) | 2.19 |
| GAS5 | NR_002578 | Growth arrest-specific 5 | 2.18 |
| ATP6AP1L | NM_001017971 | ATP6AP1L ATPase, H+ transporting, lysosomal accessory protein 1-like | 2.18 |
| C2orf74 | BC014578 | Hypothetical gene supported by AK075484 | 2.17 |
| CIRBP | AK128423 | cDNA FLJ46566 fis, clone THYMU3040829, moderately similar to Cold-inducible RNA-binding protein | 2.17 |
| C19orf18 | NM_152474 | Chromosome 19 open reading frame 18 | 2.16 |
| SEC63D1 | NM_198550 | SEC63 domain containing 1 | 2.14 |
| C1orf97 | ENST00000367003 | C1orf97 chromosome 1 open reading frame 97 | 2.12 |
| CHEK1 | BX419129 | CHEK1 CHK1 checkpoint homolog (S. pombe) | 2.11 |
| ENST00000343253 | ENST00000343253 | Intron CCDC18 coiled-coil domain containing 18 | 2.11 |
| CCT6B | NM_006584 | Chaperonin containing TCP1, subunit 6B | 2.11 |
| LOC149134 | NM_207326 | Hypothetical protein LOC149134 | 2.10 |
| ZNF688 | NM_145271 | Zinc finger protein 688 | 2.10 |
| SAMD13 | NM_001010971 | Sterile alpha motif domain containing 13 | 2.10 |
| ENST00000339446 | ENST00000339446 | Hypothetical LOC387763, mRNA (cDNA clone IMAGE:6272440), partial cds | 2.09 |
| RCBTB2 | NM_001268 | Regulator of chromosome condensation (RCC1) and BTB (POZ) domain containing protein 2 | 2.09 |
| KIAA1908 | AB067495 | Hypothetical protein LOC114796 | 2.09 |
| ADRA1B | NM_000679 | Adrenergic, alpha-1B-, receptor | 2.08 |
| TCAM1 | NR_002947 | Testicular cell adhesion molecule 1 homolog (mouse) | 2.08 |
| NPAL2 | AK025015 | cDNA: FLJ21362 fis, clone COL02886 | 2.08 |
| C21orf6 | NM_016940 | Chromosome 21 open reading frame 6 | 2.08 |
| MSTP9 | NR_002729 | Macrophage stimulating, pseudogene 9 | 2.08 |
| TncRNA | NR_002802 | Trophoblast-derived noncoding RNA | 2.08 |
| LOC643684 | XM_931745 | Hypothetical protein LOC643684 | 2.07 |
| ADAM12 | NM_003474 | ADAM metallopeptidase domain 12 (meltrin alpha) | 2.07 |
| PPP1R15A | NM_014330 | Protein phosphatase 1, regulatory (inhibitor) subunit 15A | 2.07 |
| ADAMTS13 | NM_139025 | ADAM metallopeptidase with thrombospondin type 1 motif, 13 | 2.07 |
| SYTL1 | NM_032872 | Synaptotagmin-like 1 | 2.06 |
| ZC3H6 | AK131416 | cDNA FLJ16526 fis, clone OCBBF2006987 | 2.06 |
| WDR78 | NM_207014 | WD repeat domain 78 | 2.06 |
| ARHGEF2 | NM_004723 | Rho/rac guanine nucleotide exchange factor (GEF) 2 | 2.05 |
| PRIM1 | NM_000946 | Primase, polypeptide 1, 49kDa | 2.05 |
| DKFZp762P2111 | AK022976 | ZNF783 zinc finger family member 783 | 2.05 |
| C22orf23 | NM_032561 | Chromosome 22 open reading frame 23 | 2.04 |
| ABHD1 | BC028378 | Abhydrolase domain containing 1 | 2.04 |
| AF075112 | AF075112 | L1ME4 repeat | 2.04 |
| AX721087 | AX721087 | SCXA scleraxis homolog A (mouse) AND SCXB scleraxis homolog B (mouse) | 2.02 |
| THC2326212 | THC2326212 | LOC399815 chromosome 10 open reading frame 88 pseudogene | 2.02 |
| SMPX | NM_014332 | Small muscle protein, X-linked | 2.01 |
| LOC388335 | NM_001004313 | Similar to RIKEN cDNA A730055C05 gene | 2.01 |
| LOC285033 | CR619653 | Hypothetical protein | 2.00 |
| TSLP | NM_033035 | Thymic stromal lymphopoietin | 2.00 |


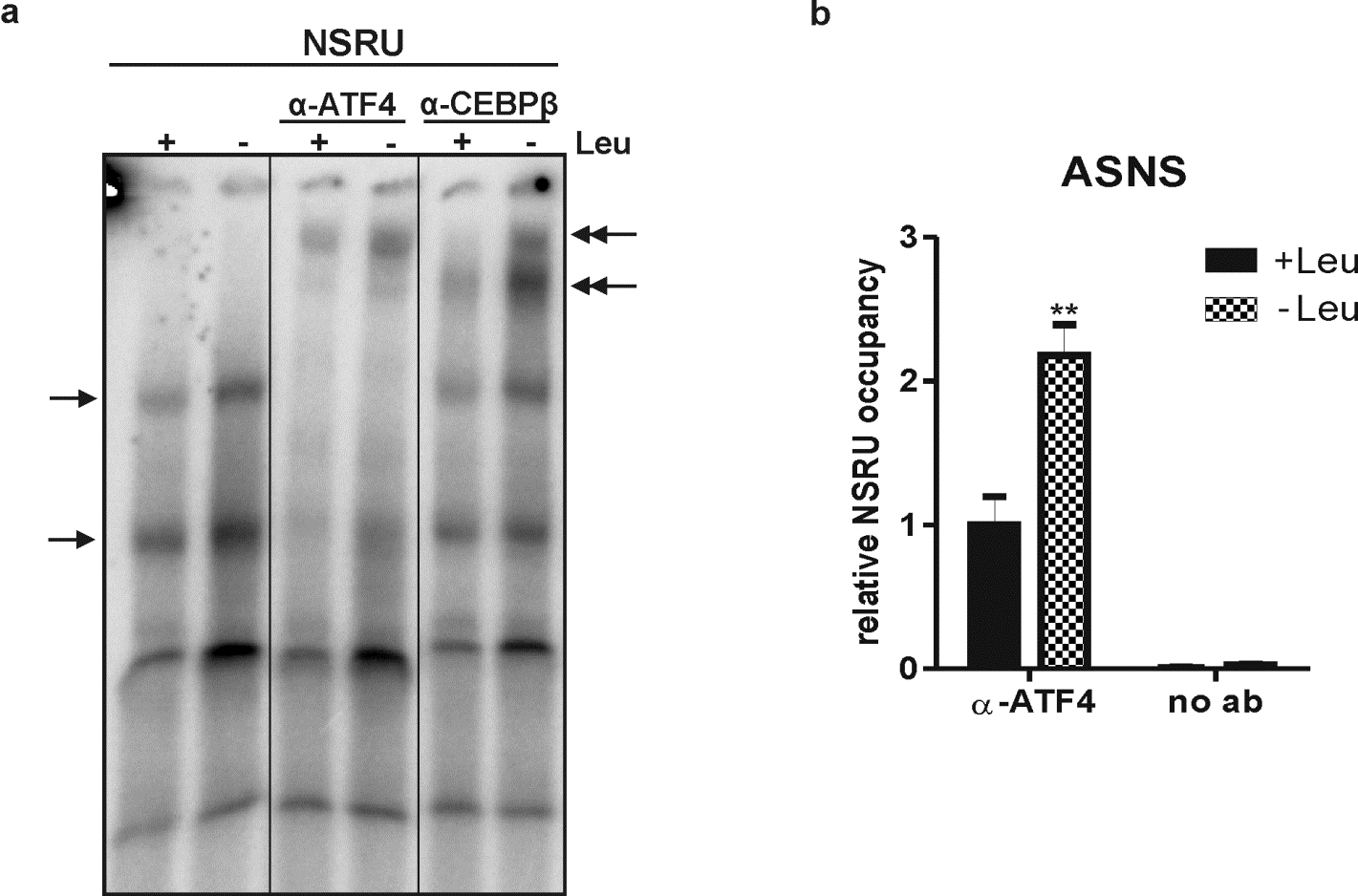


**Fig. S1** EMSA and ChIP for the NSRU of the ASNS promoter. **a** An electrophoretic mobility shift assay was performed with extracts of leucine deprived HEK293 cells (as used in Fig. 1D) and a double-stranded oligonucleotide for the NSRU sequence. Supershifts were induced with a rabbit polyclonal ATF4 antibody (sc-200; Santa Cruz; 1:1000) and a rabbit polyclonal C/EBPβ antibody (sc-150; Santa Cruz; 1:1000). Single arrows indicate the primary complexes formed, double arrows indicate the supershifted complexes. **b** Chromatin immunoprecipitation was performed (as in Fig 1e) using an anti-ATF4 antibody. Bound chromatin was analyzed by QPCR using a primer set surrounding the NSRU of the ASNS promoter. As a control the ChIP was performed without an antibody. Error bars represent SD; ***P*<0.01, relative to +Leu


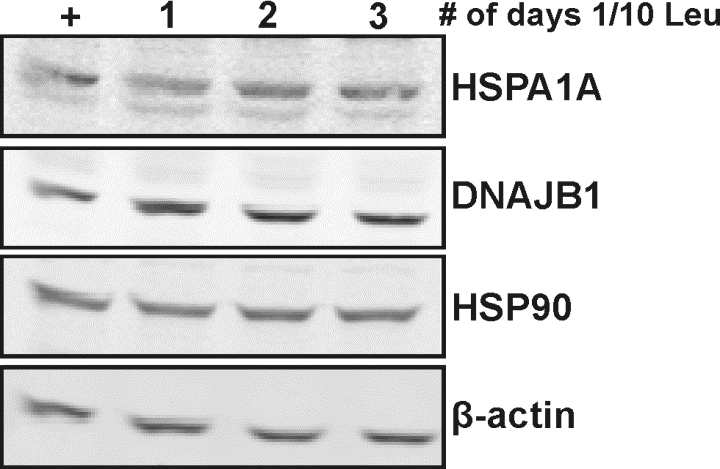


**Fig. S2** Heat shock protein levels in cells cultured with limited leucine. HEK293 cells were cultured for the indicated times in medium containing 45 µM leucine. As a control, cells were cultured in parallel in standard medium containing 450 µM leucine (+). Medium was changed every day. Lysates were subjected to SDS-PAGE and western blot analysis using antibodies against the indicated proteins


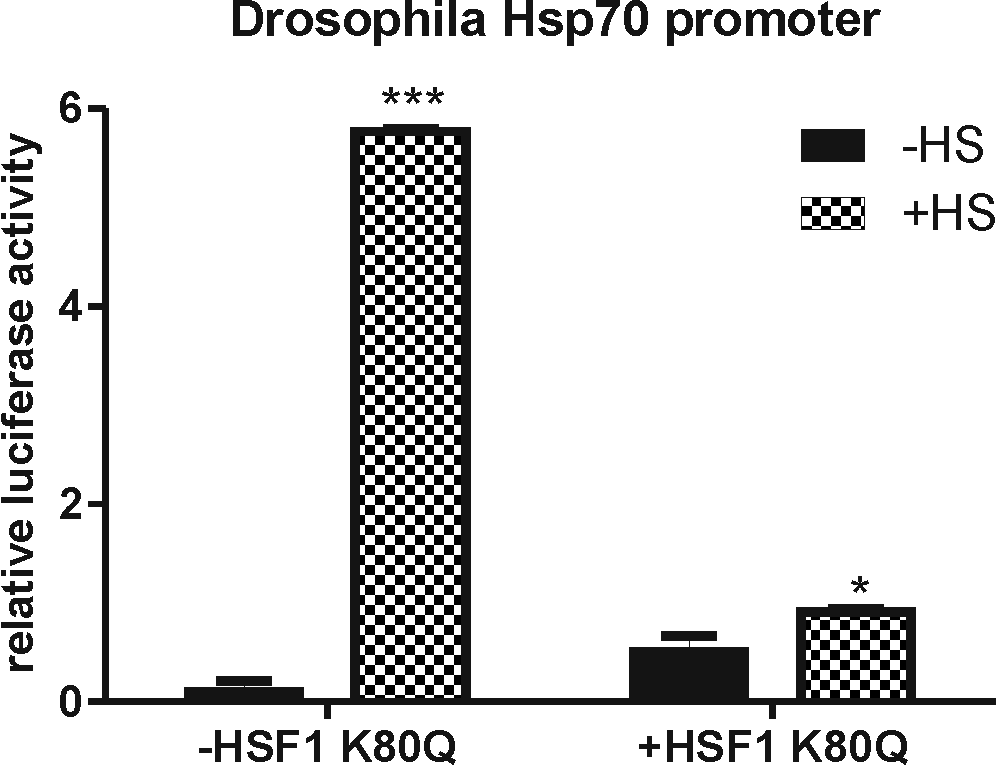


**Fig. S3** HSF1 K80Q mutant. HEK-HSF1K80Q cells were transfected with the *Drosophila melanogaster* Hsp70-luciferase reporter plasmid (Doerwald et al. (2003), J. Biol. Chem. **278,** 49743-49750) and cultured for 48 h in the presence or absence of doxycycline. Cells were exposed to a heat shock for 30’ at 45°C or left at 37°C. Six hours after recovery, cells were harvested and assayed for reporter gene activities. The results are the average of three independent transfections. Error bars represent SD; **P*<0.05; ****P*<0.001


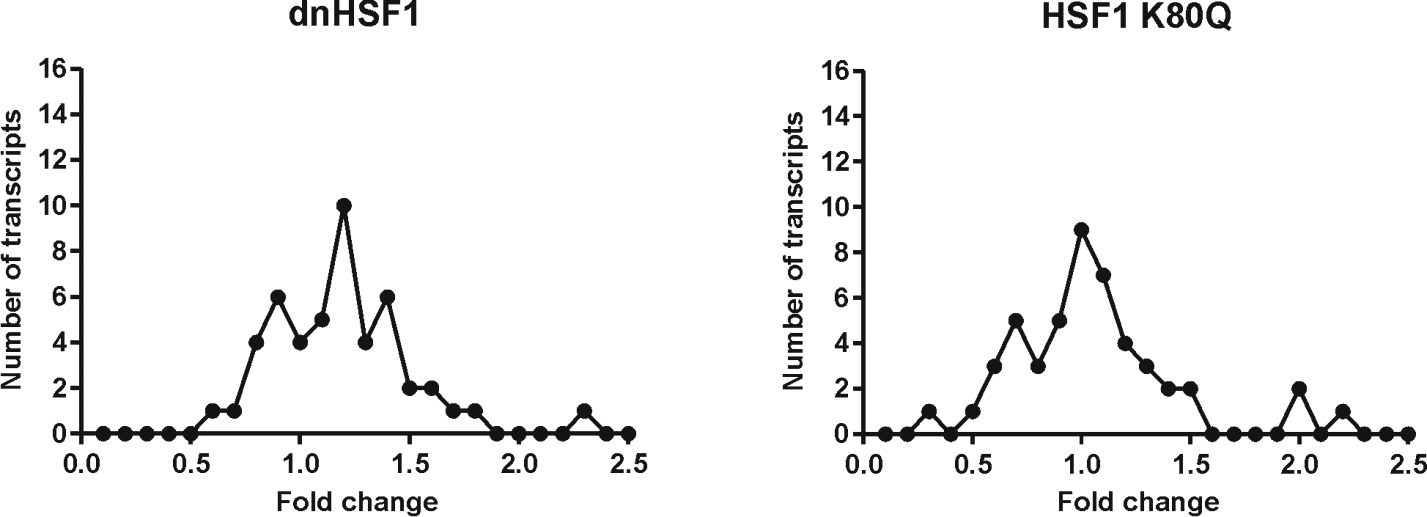


**Fig. S4** The levels of amino acid responsive gene transcripts are affected by the inactivation of HSF1. Histograms were made of the fold change in the level of amino acid responsive transcripts upon expression of dnHSF1 or HSF1 K80Q (compared to overexpression of wtHSF1)


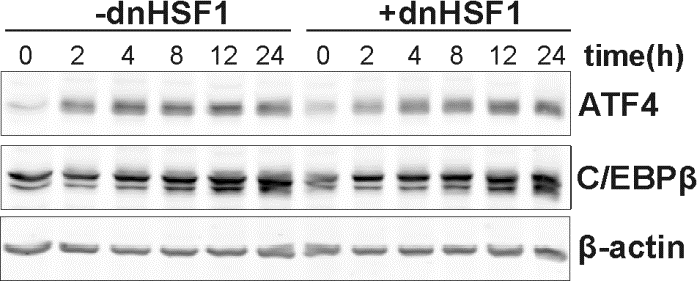


**Fig. S5** ATF4 and C/EBPβ expression levels upon leucine starvation are not affected by dnHSF1 expression. HEK-dnHSF1 cells were left untreated or expression of dnHSF1 was induced. At 24 h after induction, cells were deprived of leucine and harvested at the indicated time points. Cell lysates were subjected to SDS-PAGE and western blot analysis using the antibodies against the indicated proteins [rabbit polyclonal ATF4 antibody (sc-200; Santa Cruz; 1:1000), rabbit polyclonal C/EBPβ antibody (sc-150; Santa Cruz; 1:1000), and mouse monoclonal β-actin antibody (AC-15; Sigma; 1:5000)
